# Comparative Clinical Outcomes Across Ambulatory Cardiac Monitoring Modalities

**DOI:** 10.1002/clc.70500

**Published:** 2026-10-16

**Authors:** Suneet Mittal, Matthew R. Reynolds, Alejandro Hughes, Erin K. Buysman, Erik M. Hendrickson, Ken Boyle, Brent Wright, Jeffrey M. Ashburner, Mintu P. Turakhia, Rod S. Passman

**Affiliations:** ^1^ Valley Health System Paramus New Jersey USA; ^2^ Lahey Hospital & Medical Center Burlington Massachusetts USA; ^3^ Optum Eden Prairie Minnesota USA; ^4^ iRhythm Technologies San Francisco California USA; ^5^ Stanford University Stanford California USA; ^6^ Northwestern University School of Medicine Chicago Illinois USA

**Keywords:** ambulatory cardiac monitoring, arrhythmia diagnosis, comparative effectiveness, health care utilization, long‐term continuous monitoring

## Abstract

**Background:**

Comparative effectiveness data across ambulatory cardiac monitoring (ACM) modalities are limited.

**Objective:**

To confirm and extend comparative findings across ACM modalities and vendors in commercially insured and Medicare Advantage beneficiaries without prior arrhythmia.

**Methods:**

Retrospective study using Optum Research Database. Adults ≥ 18 years with first ACM use between January 1, 2017 and June 30, 2022 were categorized as long‐term continuous monitors (LTCM), Holter, external ambulatory event monitors (AEM), or mobile cardiac telemetry (MCT). Outcomes were arrhythmia diagnosis (90 days), repeat monitoring (180 days), cardiovascular events, hospitalization, emergency department [ED] visits, and costs (365 days). Inverse probability of treatment weighting used a specific LTCM (iRhythm Technologies, San Francisco, CA) as reference.

**Results:**

Among 352,782 patients (mean age 62.0 ± 17.1 years; 61% female), 25% received LTCM, 37% Holter, 20% AEM, and 18% MCT. Among LTCM users, 58% received iRhythm LTCM. Compared with this LTCM, Holter, MCT, and other LTCM had lower odds of arrhythmia diagnosis (IPTW‐weighted ORs 0.50−0.95; *p* < 0.01); AEM did not differ. Repeat monitoring was higher for other LTCM, AEM, and Holter (IPTW‐weighted ORs 1.99−2.77; *p* < 0.001); MCT did not differ. All other modalities had higher IPTW‐weighted odds of cardiovascular events (ORs 1.10−1.66; *p* < 0.001), hospitalization (ORs 1.12−2.04; *p* < 0.001), ED visits (ORs 1.09−2.08; *p* < 0.001), and costs (IPTW‐weighted cost ratios 1.03−1.28; *p* < 0.01).

**Conclusions:**

Outcomes differed across ACM modalities and vendors, confirming observations in other payer populations. A specific LTCM was associated with higher claims‐based arrhythmia yield and lower repeat monitoring than most alternatives, and with lower hospitalization, health care utilization, and costs.

## Graphical abstract

In >350 000 commercially insured and Medicare Advantage patients undergoing ambulatory cardiac monitoring, outcomes varied across modalities and vendors. A specific long‐term continuous monitor was associated with generally higher claims‐based arrhythmia yield and lower repeat monitoring than most alternatives, as well as lower hospitalization, health care utilization, and costs.

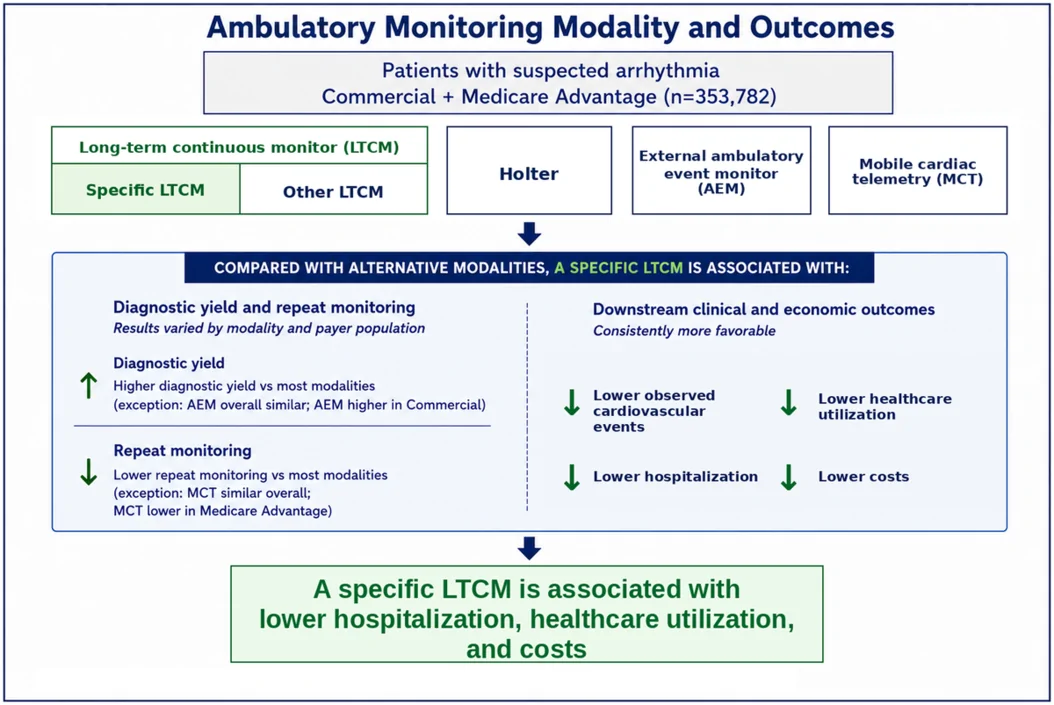

## 1 Introduction

Cardiac arrhythmias are common and increase in prevalence with age [1, 2]. Undiagnosed or untreated arrhythmias are associated with significant morbidity, mortality, and health care resource utilization in the United States [3, 4, 5, 6].

Ambulatory cardiac monitoring (ACM) plays a central role in the detection and diagnosis of arrhythmias [7, 8]. Available monitoring strategies vary widely, including short‐term (≤ 48 h) Holter monitors, long‐term (≥48 h to 14 days) continuous monitors (LTCM), event‐based external ambulatory event monitors (AEM; up to 30 days), often referred to as event‐triggered or auto‐triggered loop recorders, and mobile cardiac telemetry (MCT; up to 30 days). These modalities differ not only in monitoring duration but also in device design (e.g., lead‐based or patch‐based), continuity of recording, transmission capabilities, patient adherence, and arrhythmia detection algorithms [8, 9, 10].

Despite the prominent role of ACM in clinical practice, uncertainty remains regarding optimal modality selection. Although professional society guidelines have increasingly provided disease‐specific frameworks for ACM use, they do not specify preferred monitoring modality, duration, or vendor [7, 11], in part due to limited comparative outcomes data.

Prior studies in Medicare fee‐for‐service and selected commercial populations have demonstrated meaningful differences in diagnostic yield, need for repeat monitoring, and downstream health care utilization across ACM modalities and vendors, including differences among long‐term continuous monitoring platforms [12, 13]. The present study was designed as a confirmatory extension of those analyses in a broader, contemporary cohort that includes commercially insured and Medicare Advantage beneficiaries evaluated within a common analytic framework. The study also assessed whether prior associations were reproducible across payer groups and whether outcomes differed across LTCM platforms. Accordingly, we sought to confirm and extend prior comparative findings in diagnostic, utilization, and economic outcomes across ACM modalities and vendors.

## 2 Methods

### 2.1 Data Source

The EXploration of Cardiac AmbuLatory Monitoring in US Beneficiaries with Undiagnosed aRrhythmias (EXCALIBUR) study is a retrospective cohort study of ACM in routine practice using the Optum Research Database, a large, de‐identified administrative claims repository integrating enrollment, pharmacy, and medical claims (Optum, Eden Prairie, MN). The data source includes commercially insured and Medicare Advantage beneficiaries from across the United States and provides broad geographic, demographic, and socioeconomic representation.

The data source contains comprehensive longitudinal information including demographics, inpatient and outpatient medical encounters, International Classification of Diseases, Tenth Revision, Clinical Modification (ICD‐10‐CM) diagnoses, Current Procedure Terminology (CPT) codes, outpatient prescription dispensations, and mortality. This breadth allows for characterization of baseline risk factors, longitudinal follow‐up, and capture of health care utilization and cost outcomes. The Optum Research Database has been widely used in peer‐reviewed outcomes research, providing a reliable lens into real‐world practice outside the Medicare Fee‐for‐Service environment [12, 13]. The Optum Research Database is a de‐identified research database that contains commercial and Medicare Advantage claims, containing both medical and pharmacy information. The use of de‐identified claims data from the Optum Research Database for retrospective research studies was reviewed by a centralized institutional review board (WCG Clinical, Cary, NC) and granted an IRB exemption under 45 CFR § 46.104(d)(4). Informed consent was not required.

### 2.2 Study Population

The study observation period spanned January 1, 2016 through December 31, 2022. This window allowed for assessment of baseline comorbidities in the 12 months prior to index ACM, a minimum 6‐month follow‐up period, and evaluation of contemporary monitoring practices during an era when patch‐based monitors were widely adopted. During this period, the data source represented approximately 8% of commercially insured and 20% of Medicare Advantage beneficiaries nationwide, enabling analyses across populations underrepresented in prior studies.

Cohort construction mirrored the approach used in prior Medicare Fee‐for‐Service and commercial insurance analyses [14, 15]. We identified patients ≥18 years with their first ACM claim between January 1, 2017 and June 30, 2022. In fee‐for‐service systems, ACM claims use a split billing system in which the technical component is billed by the integrated diagnostic testing facility and the professional component is billed by the interpreting physician. Since professional claims may lag relative to the technical fee claim, the index date was defined as the date of the technical component claim. Patients with more than one ACM claim on the index date were excluded.

We constructed a new disease cohort (“diagnosis‐naïve”) by excluding patients with any evidence of a prior arrhythmia diagnosis, arrhythmia‐related procedure, or prescription for antiarrhythmic therapy during the 12‐month baseline period preceding the index date. Disqualifying procedures included catheter ablation of arrhythmia, direct‐current cardioversion, pacemaker or defibrillator implantation, cardiac resynchronization therapy, and cardiac surgery. Continuous enrollment in medical and pharmacy benefits for ≥12 months before and ≥6 months after the index date was required to ensure adequate capture of baseline comorbidities, medications, and procedures. Follow‐up duration beyond 6 months varied across patients based on available enrollment. The specific clinical indication for ACM and symptom status were not available in claims. However, because screening is not a covered indication for ACM by commercial insurance or Medicare fee‐for‐service and systematic screening was not performed by Medicare Advantage health systems during the study period, the study population primarily reflects patients undergoing ACM for symptoms or clinical suspicion of arrhythmia.

### 2.3 Monitoring Strategies

Eligible patients were categorized into mutually exclusive ACM cohorts, based on CPT technical component codes: long‐term continuous monitoring (LTCM [0297T, 93243, 93247]), Holter monitors (93226), external ambulatory electrocardiogram monitors (AEM [93271]), and mobile cardiac telemetry (MCT [93229]). Within the LTCM category, patients were further classified as receiving a specific LTCM (iRhythm Technologies, San Francisco, CA) or other LTCM devices based on the National Provider Identifier (NPI) of the parent integrated diagnostic testing facility associated with the billing claim.

Because LTCM is not a homogeneous category and prior comparative‐effectiveness studies found that the iRhythm platform was associated with higher claims‐based diagnostic yield and lower repeat monitoring than other LTCM platforms, the prespecified analysis evaluated iRhythm separately and used it as the reference group rather than pooling all LTCM devices [14, 15].

### 2.4 Baseline Characteristics

Patient demographics included age, sex, race/ethnicity, geographic region, and insurance type. Clinical history was assessed during the 12‐month baseline period. Baseline comorbidities were identified using the top 20 comorbid conditions from the Agency for Health care Research and Quality Clinical Classification Software (AHRQ CCS) and supplemented with prespecified cardiovascular and arrhythmia‐related comorbidities relevant to monitor selection and outcomes (Supporting Information S1: Table 1). These variables were selected to support comprehensive characterization of baseline health status and adjustment for differences between monitoring cohorts. Overall comorbidity burden was summarized using the Quan–Charlson Comorbidity Index. These covariates were selected to allow comprehensive adjustment for baseline health status and to contextualize differences between Commercial and Medicare Advantage enrollees.

### 2.5 Outcomes

We evaluated the following outcomes:

- Arrhythmia diagnosis yield. The primary outcome was a new diagnosis of atrial fibrillation, atrial flutter, atrioventricular block, supraventricular tachycardia, paroxysmal tachycardia, ventricular tachycardia, or ventricular flutter within 90 days of the index ACM. Diagnoses were ascertained from medical claims using prespecified ICD‐10‐CM codes (Supporting Information S1: Table 2). These arrhythmias were selected because they represent clinically actionable diagnoses and comprise the arrhythmia subset included in the Centers for Medicare & Medicaid Services (CMS) Hierarchical Condition Category framework, a diagnosis‐based classification system used for risk adjustment and prediction of health care expenditures. A 90‐day window was selected to capture diagnoses temporally associated with the monitoring episode while minimizing unrelated incident diagnoses.
- Repeat monitoring. Repeat ACM testing was defined as any additional ACM claim occurring 180 days after index. Claims for the same modality within 7 days of index were considered part of the initial monitoring episode to account for administrative rebilling or extended wear. Claims beyond 7 days or for a different modality were classified as retesting. This definition aligns with prior Medicare analyses to maintain comparability across studies.
- Cardiovascular events. We assessed ischemic stroke, systemic embolism, myocardial infarction, syncope, and cardiac arrest during the first year following index ACM use. Events were identified using prespecified ICD‐10‐CM code lists and selected to reflect arrhythmia‐related complications.
- Health care utilization. All‐cause inpatient hospitalizations and emergency department (ED) visits were assessed during the first year following index ACM use
- Health care costs. All‐cause health care costs reflected total health plan and patient paid amounts. Costs during follow‐up were calculated as the sum of insurer payments, patient copayments, and coinsurance, adjusted to 2022 US dollars using the medical care component of the Consumer Price Index. To account for variable follow‐up times, costs were annualized and reported as per‐patient‐per‐year (PPPY).

### 2.6 Statistical Analysis

Analyses were performed for the overall population and stratified by insurance type (Commercial vs. Medicare Advantage).

Descriptive statistics were reported as means with standard deviations, medians with interquartile ranges, and percentages. Between‐group comparisons were assessed with *t*‐tests for continuous variables and *χ* ^2^ tests for categorical variables.

Inverse probability of treatment weighting (IPTW) was used to control for potential confounding of the association between the outcomes and ACM modality. Propensity scores were estimated from multinomial logit models incorporating demographic, clinical, and medication covariates. Patients were weighted by the inverse probability of receiving their observed ACM modality. Covariate balance was assessed using standardized mean differences, with absolute values < 0.10 considered acceptable.

IPTW models were estimated separately within Commercial and Medicare Advantage populations, and weighted estimates were combined to generate overall population estimates (Commercial + Medicare Advantage).

Time‐to‐event outcomes were evaluated using IPTW‐weighted Kaplan−Meier methods. Odds ratios (ORs) and 95% confidence intervals for binary outcomes were derived from weighted, censor‐adjusted estimates. Health care costs were analyzed using IPTW‐weighted generalized linear models with a log link and gamma distribution.

A sensitivity analysis was conducted for the arrhythmia diagnosis outcome excluding patients with an inpatient admission or ED visit prior to the first observed arrhythmia diagnosis during follow‐up, to account for potential ascertainment bias related to acute care encounters.

All analyses were performed using SAS 9.4 (SAS Institute; Cary, NC). Two‐sided *p* values <0.05 were considered statistically significant. All data management and statistical analyses were performed by Optum using a prespecified statistical analysis plan. Neither iRhythm Technologies nor its employees had access to the underlying dataset or conducted any analyses. All authors had access to the statistical outputs and results generated from these analyses.

## 3 Results

### 3.1 Combined Cohort

#### 3.1.1 Population Characteristics

We identified 352 782 adults without an arrhythmia diagnosis in the preceding 12 months who initiated ACM during the study period (Figure 1). Device distribution was 25% LTCM (*n* = 88 057), 37% Holter (*n* = 130 500), 20% external AEM (*n* = 69 578), and 18% MCT (*n* = 64 647). Within the LTCM category, 58% received a specific LTCM (*n* = 51 320) and 42% other LTCM devices (*n* = 36 737).

**Figure 1:**
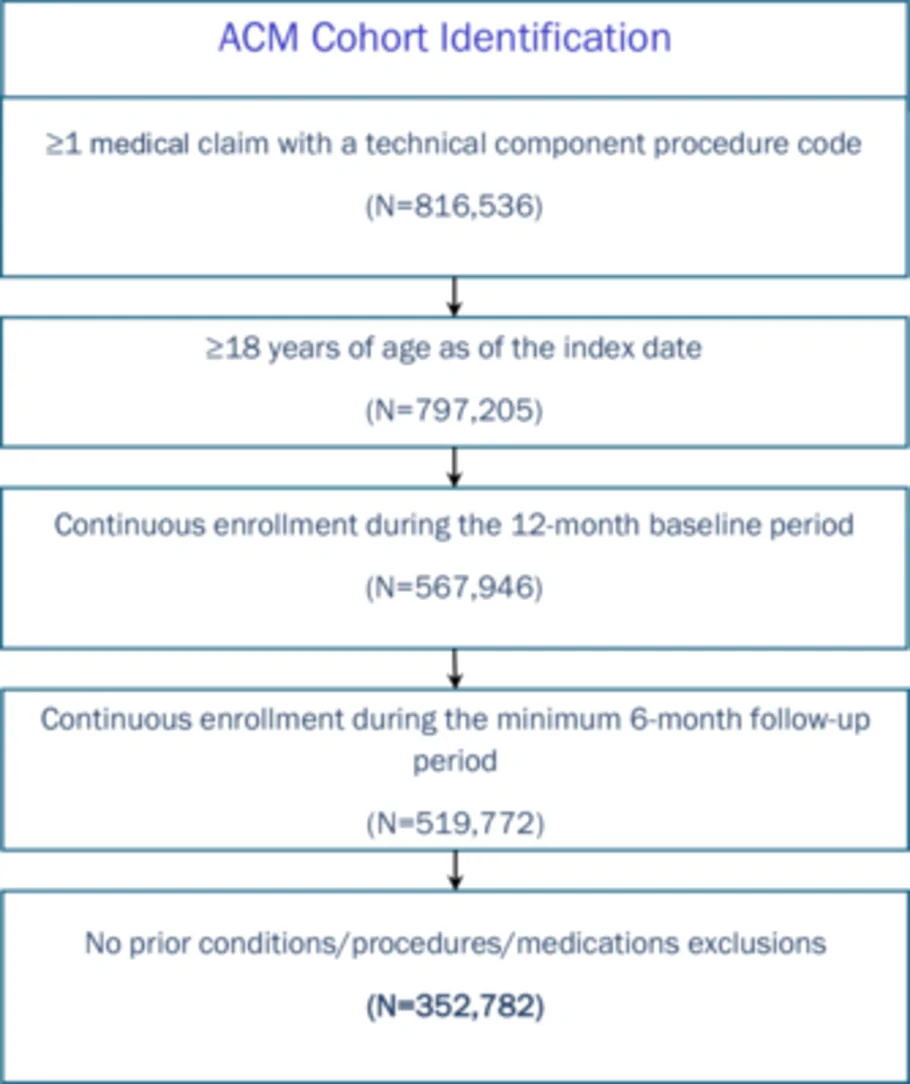
Study Cohort Selection. Flow diagram of study cohort selection from the Optum Research Database. Adults with ≥ 1 ambulatory cardiac monitoring (ACM) claim were filtered by age ≥ 18 years, continuous enrollment, and absence of prior arrhythmia diagnoses, procedures, or antiarrhythmic therapy. The final cohort included 352 782 patients undergoing first‐time ACM.

Prior to weighting, median age ranged from 58 (IQR 45–70) years among AEM users to 72 (IQR 66–78) years among MCT users; the proportion aged ≥ 65 years varied widely from 35.8% in the AEM cohort (which was 64.6% commercially insured, 35.4% Medicare Advantage) to 78.8% in the MCT cohort (14.0% commercially insured, 86.0% Medicare Advantage). Women comprised 60%–62% of all groups. The distribution of comorbidities differed substantially by monitoring modality. AEM users exhibited the lowest prevalence of hypertension, diabetes, and heart failure, consistent with their younger age and lower risk profile. By contrast, MCT patients had the most comorbidities, including the highest rates of heart failure, diabetes, chronic kidney disease, and sleep apnea, highlighting important baseline differences in case mix across monitoring cohorts (Table 1).

**Table 1:** Unweighted baseline patient and clinical characteristics, with standardized mean differences after IPTW‐weighting, for the overall combined Commercial and Medicare Advantage population.

Characteristic	iRhythm LTCM	non‐iRhythm LTCM	Holter	AEM	MCT	Maximum weighted SMD
Total (*N*)	51 320	36 737	130 500	69 578	64 647	
Age, years, median (IQR)	65 (49–74)	66 (50–74)	66 (49–74)	58 (45–70)	72 (66–78)	
Age group, years, %						
18–44	19.4	19.0	19.7	25.0	5.4	0.005
45–64	29.7	29.1	28.4	39.3	15.9	0.024
65+	50.9	51.9	52.0	35.8	78.8	0.018
Sex, %						
Female	61.5	61.7	60.2	61.0	62.2	0.008
Male	38.5	38.3	39.8	39.1	37.9	0.008
Race/ethnicity, %						
Hispanic	7.3	9.6	6.9	7.7	8.7	0.010
Non‐Hispanic White	72.8	69.6	75.1	73.3	68.2	0.011
Non‐Hispanic Black	9.1	11.1	10.1	10.4	15.3	0.004
Non‐Hispanic Asian	3.9	2.8	2.2	2.3	2.4	0.013
Other/unknown	6.9	6.9	5.8	6.3	5.5	0.001
Insurance type, %						
Commercial	48.3	45.5	46.7	64.6	14.0	
Medicare	51.7	54.5	53.3	35.4	86.0	
Region, %						
Northeast	12.3	9.6	14.0	10.8	13.5	0.022
Midwest	27.3	22.5	36.8	31.5	19.4	0.014
South	40.5	51.1	39.0	45.1	55.5	0.008
West	19.9	16.8	10.3	12.6	11.6	0.012
Baseline CCI score, median (IQR)	0.00 (0.00–2.00)	0.00 (0.00–2.00)	0.00 (0.00–2.00)	0.00 (0.00–1.00)	1.00 (0.00–2.00)	
Baseline comorbid conditions, %						
Chronic kidney disease	13.2	14.2	13.1	10.6	21.8	0.005
Chronic obstructive pulmonary disorder (COPD)	9.1	9.6	9.6	7.7	16.0	0.011
Diabetes	21.5	23.9	22.0	20.0	34.2	0.008
Dyslipidemia	58.7	59.1	57.6	49.2	77.0	0.007
Heart failure	7.2	7.0	6.4	5.2	12.8	0.006
Hypertension	59.2	60.4	60.2	51.8	79.7	0.008
MI/Coronary artery disease/Atherosclerosis	23.4	22.8	21.5	17.8	37.2	0.012
Diabetes, Type 2	21.3	23.7	21.8	19.8	34.0	0.008
Any valvular disease	18.1	14.6	16.3	11.6	26.7	0.009
Stroke or transient ischemic attack						
Recent stroke (< 30 days)	8.9	4.5	3.6	10.8	17.1	0.024
Prior stroke (≥ 30 days to 1 year)	6.5	4.7	3.9	5.8	11.6	0.016
Peripheral artery disease	5.5	5.9	5.3	4.2	10.2	0.010
Sleep apnea	14.2	14.2	13.6	12.8	17.3	0.011
Syncope	20.0	16.1	16.0	19.6	29.4	0.019
Cardiomyopathy	3.2	2.7	3.1	2.1	3.8	0.008
Baseline all‐cause utilization						
Inpatient stay	13.1	10.5	9.9	11.3	22.3	0.012
Emergency department visit	43.9	36.5	38.3	35.8	54.2	0.015
Baseline all‐cause health care costs, 2022 USD, median (IQR)	7,316 (3572–16 073)	6,133 (2753–14 201)	5,922 (2720–13 270)	5,900 (2296–14 546)	9,406 (4801–20 037)	0.021

*Note:* Values shown represent unweighted baseline characteristics. Standardized mean differences (SMDs) reflect post‐IPTW balance for variables included in the weighting model.

### 3.2 Cohort Balance

Following IPTW, covariate balance between cohorts was achieved for individual baseline demographics and comorbidities, with most standardized mean differences reduced to below the 0.10 threshold (Table 1).

### 3.3 Diagnostic Yield

Within 90 days of the index monitor, the proportion of patients with a new arrhythmia diagnosis, ascertained from medical claims, differed significantly across modalities. Weighted diagnostic yield was highest for a specific LTCM (27.1%) and AEM (27.0%), followed by other LTCM (26.0%), MCT (21.5%), and Holter (15.8%). In IPTW‐weighted analyses, Holter, MCT, and other LTCM were associated with significantly lower odds of diagnosis compared with a specific LTCM (IPTW‐weighted ORs 0.50–0.95; *p* < 0.01), while AEM did not differ significantly (OR 0.99; *p* = 0.723; Table 2).

**Table 2:** Clinical outcomes among the combined commercial and medicare advantage population.

Cardiac monitor	Cohort size	New diagnosis of specified arrhythmia within 90‐days			ACM retest within 180‐days			Any cardiovascular event (excl. arrhythmia) within 365‐days		
		%	OR (95% CI)	*p* value	%	OR (95% CI)	*p* value	%	OR (95% CI)	*p* value
Monitoring Modality										
iRhythm LTCM (ref)	51 320	27.1%	—	—	3.9%	—	—	23.5%	—	—
non‐iRhythm LTCM	36 737	26.0%	0.95 (0.92−0.98)	0.002	7.6%	1.99 (1.87−2.13)	< 0.001	27.4%	1.23 (1.19−1.27)	< 0.001
Holter	130 500	15.8%	0.50 (0.49−0.52)	< 0.001	8.5%	2.27 (2.15−2.40)	< 0.001	25.2%	1.10 (1.07−1.13)	< 0.001
External AEM	69 578	27.0%	0.99 (0.97−1.02)	0.723	10.2%	2.77 (2.61−2.94)	< 0.001	33.7%	1.66 (1.61−1.71)	< 0.001
MCT	64 647	21.5%	0.74 (0.71−0.77)	< 0.001	3.9%	0.99 (0.91−1.08)	0.899	26.7%	1.19 (1.14−1.23)	< 0.001

In a sensitivity analysis excluding patients with an inpatient admission or ED visit prior to the first observed arrhythmia diagnosis, a specific LTCM was associated with higher diagnostic yield compared with all other modalities. These findings were directionally consistent across payer groups (Supporting Information S1: Table 3).

### 3.4 Repeat Monitoring

The weighted rate of retesting within 180 days was least frequent among users of a specific LTCM and MCT, and highest among AEM users. In IPTW‐weighted analyses, other LTCM, AEM, and Holter were associated with significantly higher odds of retesting compared with a specific LTCM (IPTW‐weighted ORs 1.99–2.77; all *p* < 0.001), while MCT did not differ significantly (*p* = 0.899; Table 2).

### 3.5 Cardiovascular Events

At 1‐year, the weighted composite cardiovascular event (comprising ischemic stroke, systemic embolism, myocardial infarction, syncope, and cardiac arrest) rate was lowest for a specific LTCM. IPTW‐weighted analyses showed significantly higher odds of cardiovascular events for all other device types compared with a specific LTCM (IPTW‐weighted ORs 1.10–1.66; all *p* < 0.001; Table 2).

### 3.6 Health Care Utilization

Weighted annualized hospitalization and ED visit rates at 1 year of follow‐up varied substantially by modality, with hospitalization rates ranging from 13.2% for a specific LTCM to 23.6% for AEM, and ED visit rates from 34.1% for a specific LTCM to 51.8% for AEM. In IPTW‐weighted analyses, all other modalities were associated with higher odds of hospitalization (IPTW‐weighted ORs 1.12–2.04; all *p* < 0.001) and ED utilization (IPTW‐weighted ORs 1.09–2.08; all *p* < 0.001; Table 3) at 1 year compared with a specific LTCM.

**Table 3:** Health care resource utilization and costs among the combined commercial and medicare advantage population.

Cardiac monitor	Cohort size	Emergency department visits within 365‐days			Inpatient hospital stays within 365‐days			All‐cause follow‐up medical + pharmacy costs	
		%	OR (95% CI)	*p* value	%	OR (95% CI)	*p* value	Cost ratio (95% CI)	*p* value
Monitoring Modality									
iRhythm LTCM (ref)	51 320	34.1%	—	—	13.2%	—	—	—	—
non‐iRhythm LTCM	36 737	40.6%	1.32 (1.28−1.36)	< 0.001	15.4%	1.20 (1.15−1.25)	< 0.001	1.12 (1.08−1.15)	< 0.001
Holter	130 500	36.1%	1.09 (1.07−1.12)	< 0.001	14.6%	1.13 (1.09−1.17)	< 0.001	1.03 (1.01−1.06)	0.007
External AEM	69 578	51.8%	2.08 (2.02−2.14)	< 0.001	23.6%	2.04 (1.97−2.11)	< 0.001	1.28 (1.24−1.31)	< 0.001
MCT	64 647	37.1%	1.14 (1.10−1.18)	< 0.001	14.5%	1.12 (1.07−1.18)	< 0.001	1.06 (1.02−1.11)	0.003

### 3.7 Health Care Costs

Weighted median annualized baseline costs ranged from $6799 (other LTCM) to $7254 (MCT). During follow‐up, annualized costs were lowest for users of a specific LTCM ($8672) and highest for AEM users ($12 047). The increase in median annualized costs from baseline to follow‐up was smallest for a specific LTCM ($1845), followed by MCT ($1968), and largest for AEM ($5072). In IPTW‐weighted analyses, follow‐up health care costs were significantly higher for all other modalities compared with a specific LTCM (IPTW‐weighted cost ratios [RRs] 1.03–1.28; all *p* < 0.01; Table 3).

### 3.8 Subgroup Analysis: Commercially Insured Patients

Among 156 487 commercially insured patients, device distribution was 16% a specific LTCM, 11% other LTCM, 39% Holter, 29% AEM, and 6% MCT. Median age was 49 years (IQR 37–59), 61% were women, and 73% were non‐Hispanic White. Hypertension (38%) and dyslipidemia (37%) were the most common comorbidities (Supporting Information S1: Table 4).

In commercially insured patients, findings differed from the overall cohort for diagnostic yield. AEM was associated with higher diagnostic yield compared with a specific LTCM, while Holter and MCT were lower and other LTCM did not differ significantly. In sensitivity analyses excluding patients with acute care encounters prior to diagnosis, these differences were attenuated, with AEM no longer differing from a specific LTCM (Supporting Information S1: Table 3). A specific LTCM was associated with lower repeat monitoring, lower observed cardiovascular event rates, fewer hospitalizations and ED visits, and lower costs than all other modalities (Supporting Information S1: Tables 5 and 6).

### 3.9 Subgroup Analysis: Medicare Advantage Patients

Among 196,295 Medicare Advantage beneficiaries, 14% received a specific LTCM, 10% other LTCM, 35% Holter, 13% AEM, and 28% MCT. Median age was 73 years (IQR 69–79), 61% were women, and 72% were non‐Hispanic White. Hypertension (81%) and dyslipidemia (78%) were the most prevalent comorbidities.

Among Medicare Advantage beneficiaries, findings were generally consistent with the overall cohort. A specific LTCM was associated with higher diagnostic yield compared with all other modalities, including AEM. These findings were unchanged in sensitivity analyses excluding patients with acute care encounters prior to diagnosis (Supporting Information S1: Table 3). Compared with a specific LTCM, all other modalities were associated with higher observed cardiovascular event rates and higher rates of hospitalization and ED visits. Repeat monitoring was higher with other LTCM, Holter, and AEM but lower with MCT. Follow‐up costs were higher with other LTCM and AEM; costs did not differ significantly between the specific LTCM and Holter or MCT (Supporting Information S1: Tables 8 and 9).

## 4 Discussion

In this national analysis of over 350 000 commercially insured and Medicare Advantage beneficiaries without prior arrhythmia diagnoses, outcomes varied across ACM modalities and vendors, confirming prior observations in Medicare fee‐for‐service and commercial populations. A specific LTCM (iRhythm) was associated with generally higher claims‐based arrhythmia detection and lower repeat monitoring than most alternative strategies, lower hospitalization and ED utilization across all comparisons, and lower follow‐up costs overall. These findings extend prior comparative‐effectiveness evidence to a broader, contemporary population.

Despite substantial differences in age and comorbidity burden between Commercial and Medicare Advantage populations, patterns were broadly consistent across payer groups for repeat monitoring and downstream outcomes. There was modest variation in diagnostic yield, ascertained from ICD‐10 coding, across payer populations, with AEM demonstrating higher yield than a specific LTCM in commercially insured patients but lower yield in Medicare Advantage beneficiaries. These differences may reflect variation in clinical practice patterns, coding, or opportunities for diagnosis related to acute care encounters. In contrast, downstream clinical actions and outcomes were highly consistent across payer groups. Repeat monitoring, which is more likely driven by unresolved clinical need, was lower with a specific LTCM across most comparisons. A specific LTCM was also consistently associated with lower observed cardiovascular event rates, reduced hospital utilization, and smaller increases in health care costs compared with alternative monitoring strategies.

Our results confirm prior vendor‐ and modality‐specific analyses in Medicare fee‐for‐service and selected commercial populations while extending those observations to commercially insured and Medicare Advantage beneficiaries identified from a single claims database and evaluated using consistent cohort definitions, outcome definitions, and analytic methods [14, 15]. Although claims‐based diagnostic yield varied across payer groups, findings for repeat monitoring, hospitalization, ED utilization, and costs were broadly consistent. The reproducibility of these findings across payer environments suggests that the prior findings are not limited to a single insurance population.

Several factors may potentially contribute to the observed differences across monitoring modalities. Monitoring strategies that provide longer and continuous, uninterrupted recording, such as LTCM, may improve detection of intermittent or infrequent arrhythmias compared with shorter‐duration or intermittently recording approaches [16, 17, 18]. Differences in effective wear time may also contribute. Lead‐based Holter monitors may require removal for bathing or daily activities, while longer‐duration external monitors may require repatching, charging, or other patient interaction that can interrupt monitoring continuity. In contrast, prior studies of patch‐based long‐term continuous monitoring have demonstrated high rates of uninterrupted wear over the prescribed monitoring period [19, 20]. Although AF burden cannot be ascertained from claims data, it is increasingly recognized as an important clinical measure from extended monitoring [21]. Accurate characterization of AF burden depends on sufficient monitoring duration and continuity, further emphasizing that monitoring strategies may differ in the information they provide beyond binary arrhythmia detection. The accuracy of AF burden estimation has also been shown to vary across ACM devices [22].

Differences in patient adherence and device design may also influence effective wear time and hence diagnostic yield and downstream clinical decision‐making [23]. Higher retesting rates among Holter and AEM users may reflect a mismatch between monitoring duration and symptom frequency, suggesting potential inefficiencies in initial test selection. In addition, variation in acute care utilization may influence opportunities for arrhythmia detection independent of ambulatory monitoring. In sensitivity analyses excluding patients with inpatient admissions or ED visits prior to diagnosis, a specific LTCM was associated with higher diagnostic yield across modalities, suggesting that differences observed in the primary analysis may be influenced in part by ascertainment bias related to acute care encounters. Differences in downstream cardiovascular event rates may also reflect residual confounding and differences in underlying patient risk that cannot be fully captured in claims‐based analyses.

These findings suggest that clinicians should consider monitoring modality and platform in relation to diagnostic performance and repeat testing, particularly because symptom frequency alone may not reliably guide optimal monitoring duration [18]. For payers and health systems, the observed associations with hospitalization, health care utilization, and costs may be relevant when evaluating monitoring strategies in value‐based care settings. Because access and reimbursement vary substantially across countries, the applicability of these U.S.‐based findings to other health care systems may depend on local coverage and reimbursement structures. In a recent analysis across seven European countries, traditional Holter monitoring was universally reimbursed, whereas newer extended‐duration technologies, including long‐term continuous monitoring, generally lacked specific reimbursement, potentially limiting clinicians' ability to select monitoring strategies based on comparative clinical evidence [24]. However, this study does not establish that monitor choice causes differences in cardiovascular events or hospitalization. Prospective studies that capture monitoring indication, actual wear and analyzable time, ECG‐confirmed arrhythmias, subsequent treatment decisions, and downstream outcomes are needed to determine whether monitoring strategy alters care pathways. The present analysis was limited to clinician‐ordered, ECG‐based ambulatory monitoring and did not capture consumer smartwatches or smartphone photoplethysmography. Use of wearable technologies for rhythm monitoring is emerging, while their integration into routine clinical pathways and impact on clinical outcomes remain active areas of development and investigation [25, 26, 27].

### 4.1 Limitations

This study has several limitations. As a retrospective analysis of administrative claims data, we lacked detailed clinical information, including monitoring indication, symptom characteristics, ECG findings, actual wear time, signal quality, and analyzable recording duration, which may influence both modality selection and outcomes. We therefore cannot distinguish the independent contribution of prescribed monitoring duration from other platform‐specific features, such as patient adherence, analyzable recording time, workflow integration, or diagnostic algorithms. Claims are generated for billing and reimbursement; arrhythmia diagnoses and cardiovascular events were not ECG‐confirmed or clinically adjudicated, and differences in coding practices or opportunities for diagnosis may affect measured yield. Although IPTW accounted for measured covariates, residual confounding and confounding by indication remain possible. Associations between monitor type and cardiovascular events, hospitalization, health care utilization, and costs should not be interpreted as causal, and downstream treatment changes were not evaluated. Although the specific clinical indication and symptom status were not available in claims, this population primarily reflects reimbursed ACM for symptoms or clinical suspicion rather than systematic screening. Accordingly, these findings may not be applicable to systematic AF screening in asymptomatic populations [28]. Finally, although the findings confirm prior analyses in other payer populations, generalizability may be limited in uninsured populations or health care systems with substantially different delivery and reimbursement structures.

## 5 Conclusions

In this broader, contemporary cohort of commercially insured and Medicare Advantage beneficiaries, outcomes varied by ACM modality and vendor, confirming prior observations in Medicare fee‐for‐service and commercial populations. A specific LTCM was associated with generally higher diagnostic yield and lower repeat monitoring compared with most alternatives, as well as lower hospitalization and ED utilization across all comparisons; follow‐up costs were also lower overall, although payer‐specific cost comparisons were not uniformly significant. These findings suggest that monitoring modality and device selection may be associated with differences in downstream clinical and economic outcomes and warrant further prospective evaluation.

## Author Contributions

All authors contributed to the conception or design of the study, acquisition, analysis, or interpretation of the data, and critical revision of the manuscript for important intellectual content. All authors reviewed and approved the final manuscript and agree to be accountable for the work.

## Conflicts of Interest

**S. Mittal:** consulting: Boston Scientific, Medtronic, and iRhythm. **M. Reynolds:** consulting: iRhythm, Medtronic. **A. Hughes:** Employee of Optum. **E. Buysman:** Employee of Optum. **E. Hendrickson:** Employee of iRhythm Technologies. **K. Boyle:** Employee of iRhythm Technologies. **B. Wright:** Employee of iRhythm Technologies. **J. Ashburner:** Employee of iRhythm Technologies. **M. Turakhia:** Equity in iRhythm, Connect America, Evidently, PocketRN, AliveCor, Hippocratic A; employee of iRhythm Technologies. **R. Passman:** Consulting fees: iRhythm, Abbott, MDT. Johnson and Johnson, CoreMap, Edgewise; Royalites: UpToDate.

## Supporting information


Supporting File

## Data Availability

The data underlying this study are proprietary administrative claims data from the Optum Research Database and are not publicly available. Access is subject to applicable licensing and data‐use agreements.
