# Supplementary material for: Comparative Clinical Outcomes Across Ambulatory Cardiac Monitoring Modalities
Source: Clin Cardiol. 2026 Oct 16;49(10):e70500. doi: 10.1002/clc.70500 (PMC13674421; doi:10.1002/clc.70500)
Supplement: Supplementary file 1 [file CLC-49-e70500-s001.docx]

**Supplemental Material:**

**Supplemental Table 1.** ICD-10-CM definitions used to identify baseline comorbid conditions

| **Baseline comorbidities** | **ICD-10-CM codes** |
| --- | --- |
| **Top 20 AHRQ comorbidities** |  |
| Diseases of the heart | I00–I09, I11, I13, I20–I51 |
| Hypertension | I10–I16 |
| Disorders of lipid metabolism | E78.x |
| Immunizations and screening for infectious disease | Z20–Z29 |
| Other lower respiratory disease | J40–J47 |
| Other connective tissue disease | M30–M36 |
| Eye disorders | H00–H59 |
| Other nutritional; endocrine; and metabolic disorders | E00–E07, E15–E16, E20–E34, E40–E68, E70–E90 |
| Non-traumatic joint disorders | M00–M25 |
| Diseases of the urinary system | N00–N39 |
| Spondylosis; intervertebral disc disorders; other back problems | M40–M54 |
| Other skin disorders | L00–L99 |
| Other nervous system disorders | G00–G99 |
| Ear conditions | H60–H95 |
| Diabetes mellitus without complication | E10.9, E11.9, E13.9 |
| Diseases of arteries; arterioles; and capillaries | I70–I79 |
| Respiratory infections | J00–J22 |
| Upper gastrointestinal disorders | K20–K31 |
| Benign neoplasms | D10–D36 |
| Other gastrointestinal disorders | K35–K92 |
| **Additional pre-specified comorbidities** |  |
| Anemia | D46.0–D46.4, D50.0–D53.9, D55.0–D59.9, D60.0–D60.9, D61.01–D61.9, D62, D63.0–D63.8, D64.0–D64.9, O90.81, O99.011–O99.019, O99.02–O99.03, R71.0 |
| Prior bleeding | I60.0–I62.9, I85.01–I85.11, I97.410–I97.418, I97.610–I97.618, I97.62, J94.2, K22.11, K25.0–K28.6, K29.01–K29.91, K31.811, K50.011–K52.11, K55.21, K57.01–K57.93, K62.5, K76.2, K91.61–K91.841, K92.0–K92.2, K94.01–K94.31, N92.3–N93.9, O03.1–O08.1, O20.8–O20.9, O44.10–O46.99, O67.0–O72.2, R04.0–R04.9, R31.0–R31.9, R58, S06.34–S06.39, S06.4X–S06.6X, S26.0–S27.2, T79.2XX, T80.0–T80.A, T81.501–T81.591, T82.837–T82.838, T83.83, T84.83, T85.83, Y62.1–Y65.0 |
| Anxiety disorder | F06.4, F10.180–F19.980, F40.00–F40.298, F40.8–F40.9, F41.0–F41.9, F93.0, F94.0 |
| Asthma | J45.20–J45.52, J45.901–J45.998, T48.6X5–T48.6X6 |
| Chronic kidney disease | E08.21–E13.29, I12.0–I13.2, M31.31, M32.15, M35.04, N01.0–N08, N11.0–N12, N13.721–N13.739, N13.8–N13.9, N14.0–N14.4, N15.0–N16, N18.1–N18.9, N19, N25.0–N25.9, N26.1–N26.9, Q60.1–Q62.0, T81.502–T81.592, T82.41–T82.49, T85.611–T85.791, T86.10–T86.19, Z48.22, Z49.01–Z49.32, Z91.15, Z94.0, Z99.2 |
| Chronic obstructive pulmonary disorder (COPD) | J41.0–J41.8, J42, J43.0–J43.9, J44.0–J44.9 |
| Diabetes | E08–E13 |
| Diabetes, Type 2 | E11.00–E11.9 |
| Dyslipidemia | E78.0–E78.6 |
| Heart failure | I09.81, I11.0, I13.0, I13.2, I50.1–I50.9 |
| MI/Coronary artery disease/Atherosclerosis | I21–I22; I25.x; I70.x |
| Any valvular disease | I05.0–I09.1, I33.0–I39, Q22.0–Q23.9, Z95.2–Z95.4, T82.01–T82.09, T82.221–T82.228, T82.6XX |
| Stroke or transient ischemic attack | I60.0–I62.9, I63.0–I63.9, I69.0–I69.98, G45.0–G46.8, G46.3–G46.7, I67.81 |
| Recent stroke (< 30 days) |  |
| Prior stroke (≥ 30 days - 1 year) |  |
| Peripheral artery disease | I70.0–I70.99 |
| Pulmonary embolism | I26.01–I26.99, I27.24, I27.82 |
| Sleep apnea | G47.30–G47.39 |
| Syncope | R55 |
| Venous thromboembolism (VTE) | I81, I82.0–I82.C29 |
| Cardiomyopathy | I42.x |

**Supplemental Table 2.** ICD-10-CM definitions used to identify arrhythmias for the 90-day diagnostic yield outcome.

| **ICD-10-CM** | **Description** |
| --- | --- |
| I44.0 | Atrioventricular block, first degree |
| I44.1 | Atrioventricular block, second degree |
| I44.2 | Atrioventricular block, complete |
| I44.30 | Unspecified atrioventricular block |
| I44.39 | Other atrioventricular block |
| I47.1 | Supraventricular tachycardia |
| I47.2 | Ventricular tachycardia |
| I47.9 | Paroxysmal tachycardia, unspecified |
| I48.0 | Paroxysmal atrial fibrillation |
| I48.11 | Longstanding persistent atrial fibrillation |
| I48.19 | Other persistent atrial fibrillation |
| I48.20 | Chronic atrial fibrillation, unspecified |
| I48.21 | Permanent atrial fibrillation |
| I48.3 | Typical atrial flutter |
| I48.4 | Atypical atrial flutter |
| I48.91 | Unspecified atrial fibrillation |
| I48.92 | Unspecified atrial flutter |
| I49.02 | Ventricular flutter |

**Supplemental Table 3.** Arrhythmia yield after excluding patients with hospital admissions or emergency department visits prior to diagnosis

**Supplemental Table 4.** Unweighted baseline patient and clinical characteristics, with standardized mean differences after IPTW-weighting, for the Commercial population.

**Supplemental Table 5.** Clinical outcomes among the Commercial population.

**Supplemental Table 6.** Healthcare resource utilization and costs among Commercial population.

**Supplemental Table 7.** Unweighted baseline patient and clinical characteristics, with standardized mean differences after IPTW-weighting, for the Medicare Advantage population.

**Supplemental Table 8.** Clinical outcomes among the Medicare Advantage population.

**Supplemental Table 9.** Healthcare resource utilization and costs among Medicare Advantage population.
